# Prognostic Significance of Lymphovascular Invasion in Radical Cystectomy on Patients with Bladder Cancer: A Systematic Review and Meta-Analysis

**DOI:** 10.1371/journal.pone.0089259

**Published:** 2014-02-21

**Authors:** Hwanik Kim, Myong Kim, Cheol Kwak, Hyeon Hoe Kim, Ja Hyeon Ku

**Affiliations:** Department of Urology, Seoul National University College of Medicine, Seoul, Korea; University of Texas MD Anderson Cancer Center, United States of America

## Abstract

**Purpose:**

The objective of the present study was to conduct a systematic review and meta-analysis of published literature to appraise the prognostic value of lymphovascular invasion (LVI) in radical cystectomy specimens.

**Materials and Methods:**

Following the PRISMA statement, PubMed, Cochrane Library, and SCOPUS database were searched from the respective dates of inception until June 2013.

**Results:**

A total of 21 articles met the eligibility criteria for this systematic review, which included a total of 12,527 patients ranging from 57 to 4,257 per study. LVI was detected in 34.6% in radical cystectomy specimens. LVI was associated with higher pathological T stage and tumor grade, as well as lymph node metastasis. The pooled hazard ratio (HR) was statistically significant for recurrence-free survival (pooled HR, 1.61; 95% confidence interval [CI], 1.26–2.06), cancer-specific survival (pooled HR, 1.67; 95% CI, 1.38–2.01), and overall survival (pooled HR, 1.67; 95% CI, 1.38–2.01), despite the heterogeneity among included studies. On sensitivity analysis, the pooled HRs and 95% CIs were not significantly altered when any one study was omitted. The funnel plot for overall survival demonstrated a certain degree of asymmetry, which showed slight publication bias.

**Conclusions:**

This meta-analysis indicates that LVI is significantly associated with poor outcome in patients with bladder cancer who underwent radical cystectomy. Adequately designed prospective studies are required to provide the precise prognostic significance of LVI in bladder cancer.

## Introduction

Urothelial carcinoma of the bladder is the fifth most common cancer worldwide, with an estimated incidence of 73,510 cases and 14,880 deaths in the United States for 2012 [1]. While the majority of patients present with non-muscle invasive lesions amenable to local resection, radical cystectomy with pelvic lymphadenectomy continues to represent the gold standard for patients with muscle invasive tumors, as well as for patients with high-risk non-muscle invasive disease. Despite recent multidisciplinary advances in its treatment, bladder cancer continues to carry unacceptably high rates of morbidity and mortality. Thus, the identification of patients at high risk of poor outcome is one of the major concerns for clinicians. New strategies, including the administration of innovative and intensive neoadjuvant/adjuvant therapies, may enable improved survival in these patients.

Lymphovascular invasion (LVI) has been documented as a poor prognostic factor in many solid organ tumors [2], [3]. In a previous study, we have also demonstrated an association between the presence of LVI and poor prognosis in upper urinary tract urothelial carcinoma [4]. The prognostic value of LVI in bladder cancer has been widely investigated, owing to the fact that this feature exhibited an increasing relevance in clinical practice. Although multiple studies have been conducted on bladder cancer patients, the prognostic significance of LVI in radical cystectomy specimens is still controversial. Therefore, we have conducted an up-to-date meta-analysis to appraise the prognostic value of LVI in bladder cancer.

## Materials and Methods

### Search Strategy

We conducted and reported this systematic review and meta-analysis following the PRISMA statement [5]. A comprehensive literature search of electronic databases PubMed, SCOPUS, and Cochrane Library were performed using the following keywords: [urinary bladder neoplasms] OR [urinary AND bladder AND neoplasms] OR [bladder AND cancer] OR [bladder cancer] AND [lymphovascular] AND [invasion]. The search concluded in June 2013, and no lower date limit was used. Searches were limited to studies published in English. Conference abstracts were not selected for analysis due to the insufficient data reported.

### Study Inclusion/Exclusion Criteria

A study was considered eligible if it met all of the following inclusion criteria: (i) the study included proven diagnosis of urothelial carcinoma; (ii) the study evaluated LVI in radical cystectomy specimens; (iii) the study considered radical cystectomy as a treatment modality; (iv) the study assessed the association between LVI and survival of patients with bladder cancer; and, (v) the study provided a hazard ratio (HR) and 95% confidence interval (CI) directly or presented the data that allows for estimation of the HR and 95% CI. Studies were excluded based on any of the following criteria: (i) review articles, letters to the editor, commentaries, or case reports; (ii) laboratory studies, such as studies on bladder cancer cell lines and animal models; and (iii) duplication of previous publications. All studies were carefully examined to avoid inclusion of duplicate data. When more than one of the same patient populations was included in several publications, only the most recent or most complete study was used to avoid duplication of information. Two reviewers (HK and MK) assessed the eligibility of the screened studies independently. Agreements were reached for discrepant opinions through discussion.

### Data Extraction

To rule out subjectivity in the data gathering and entry process, data were extracted independently by two reviewers (HK and MK) for each eligible study. The extracted data were recorded by both investigators independently in separate databases. The two completed databases were compared and discussed between the two investigators to reach a consensus. We did not contact authors of eligible studies for additional data. Pre-specified data parameters to be gathered were as follows: (i) publication data including country, first author’s last name, publication year, period of recruitment, study design, inclusion and exclusion criteria, consecutiveness of patient enrollment, definition of LVI, definition of survival, and interpretation of LVI; (ii) demographic data such as sample size, age, gender, treatment, and follow-up period; (iii) tumor data including concomitant carcinoma *in situ*, variant form, pathological T stage, tumor grade, pathological N stage, and number of lymph nodes retrieved; and (iv) statistical data including the exact data of total and exposed number of subjects in? case and control groups, as well as HRs and their CIs. Multivariate Cox hazard regression analysis data were preferred in our analysis. If this analysis was not available, we extracted univariate Cox hazard regression analysis or Kaplan-Meier survival curves with log-rank p-value of survival outcomes, instead. In studies for which clinical outcomes were estimated using both multivariate and univariate analyses, the results of the multivariate analyses were used to calculate HRs and CIs.

### Quality Assessments

Methodological assessment for each of the included studies was performed by three investigators (HK, MK, and JHK), according to three quality scales from the predefined form by De Graeff et al [6], which was adapted from REMARK (Reporting recommendations for tumor MARKer prognostic studies) [7]. The quality scale has seven criteria, and a study with a total score of 8 was considered to have the highest study quality, whereas a score of zero indicated the lowest quality.

### Statistical Analysis

We obtained the log-HRs and their 95% CIs from each study and subsequently performed the meta-analysis by a random-effect model. If HRs and the corresponding standard errors were not directly reported, they were estimated according to the available survival data by using the method reported by Palmar et al [8]. An observed HR >1 implied a worse survival for the study group with positive LVI, relative to the reference group. The impact of LVI on outcome was considered statistically significant if the 95% CI did not overlap with 1 and if p<0.05. We also performed subgroup analyses to examine if our pooled estimate of the prognostic value was influenced by publication year, region, number of patients, pathologic N stage, median follow-up, HR estimation, analysis results, and methodological quality scales. To evaluate the robustness of the combined HR and to check the stability of meta-analysis, sensitivity analyses were performed by removing one study at a time. A test of heterogeneity of the combined HRs was carried out using the Chi-square test and Higgins I-squared statistic. P<0.10 was considered to represent substantial heterogeneity between studies. I^2^>50% indicated large heterogeneity among studies, whereas I^2^ values between 25% and 50% indicated moderate heterogeneity [9]. Publication bias was evaluated using the funnel plot. The Begg’s rank correlation and Egger’s linear regression were also applied to assess the potential publication bias. The nomnial level of significance was set at 5%. All 95% CIs were two-sided. The meta-analysis was performed using Review Manager (RevMan) software version 5.0 (RevMan 5; The Nordic Cochrane Center, The Cochrane Collaboration, Copenhagen, Denmark). Publication biases were evaluated by R 2.13.0 (R Development Core Team, Vienna, Austria, http://www.R-project.org).

## Results

### Study Selection

**Figure 1:**
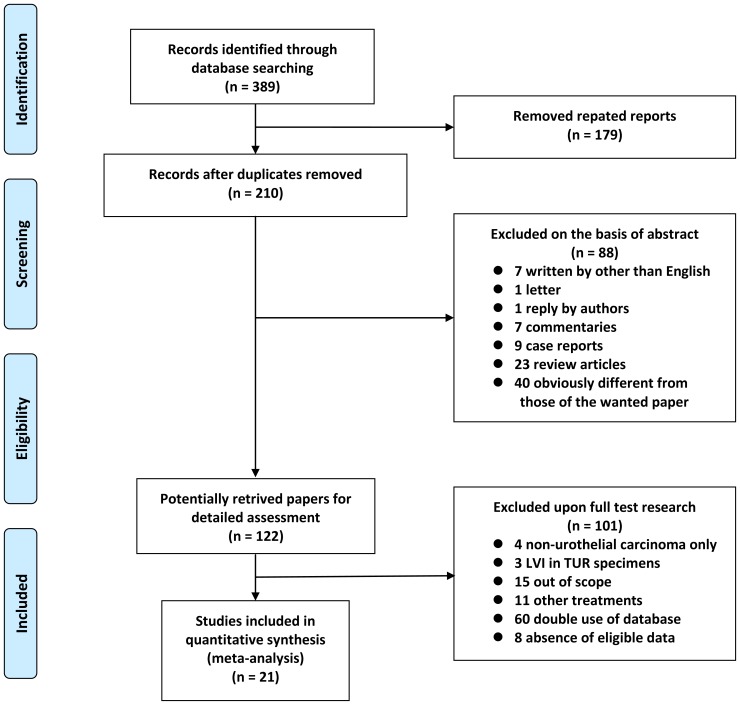
Flow chart of the literature search used in this meta-analysis.

Of the 389 articles initially identified, 179 articles were excluded as duplicate publications. After screening the titles and abstracts, an additional 88 articles were excluded. The remaining 122 articles were reviewed by full text. After the full text review, 15 were excluded because these studies did not perform survival analysis, eight were excluded because these studies did not provide sufficient data for estimation of HRs, three were excluded because LVI was not assessed for radical cystectomy specimens but for transurethral resection specimens, four were excluded because the assessment was conducted on non-urothelial carcinoma, 11 were excluded because study subjects had been treated with modalities other than radical cystectomy, and 60 were excluded for having overlapping data with another study. At the end of this culling process, 21 articles were selected for the systemic review, which included 12,527 patients, ranging from 57 to 4,257 per study [10]–[30]. Figure 1 shows a flow diagram of the selection process for relevant studies.

### Methodological Quality of the Studies

The median quality score was 3 for the 21 articles reviewed (mean: 3.6, range: 2–5) (Table 1). Ten of the included studies obtained scores of 4 or more in methodolocal assessment, indicating that they were of high quality. There was no significant correlation between study size and quality scores (Spearman’s r = −0.002, p = 0.992). There were no statistical differences of quality score according to publication year and median follow-up time. However, there was a significant difference in the quality of studies by study origin (3.2 for Asian countries vs. 4.3 for other countries, p = 0.015).

**Table 1:** Main characteristics of the eligible studies.

Study	Year	Country	Recruitmentperiod	Study design	Inclusionandexclusioncriteria	Consecutivepatients	Definitionof survival	Definitionof LVI	Interpretationof LVI	Qualityscale
Turkolmez [10]	2007	Turkey	1990–2005	Retrospective	Yes	NA	No	Yes	NA	3
Canter [11]	2008	USA	1988–2006	Retrospective	Yes	NA	No	No	NA	2
Matsumoto [12]	2008	Japan	1990–2004	Retrospective	Yes	Yes	No	Yes	Blind	5
Fairey [13]	2009	USA	1994–2007	Retrospective	Yes	Yes	Yes	No	NA	3
Streeper [14]	2009	USA	1995–2005	Retrospective	Yes	Yes	No	Yes	NA	3
Hugen [15]	2010	USA	1996–2008	Retrospective	Yes	NA	Yes	No	NA	2
Kim [16]	2010	Korea	1986–2005	Retrospective	Yes	NA	No	No	NA	3
Ku [17]	2010	Korea	1991–2000	Retrospective	Yes	Yes	Yes	Yes	NA	4
Manoharan [18]	2010	USA	1992–2008	Retrospective	Yes	NA	No	Yes	NA	2
Palmieri [19]	2010	Italy	1995–2007	Retrospective	Yes	Yes	Yes	Yes	NA	5
Shariat [20]	2010	Multination	1979–2008	Retrospective	No	NA	Yes	Yes	Blind	5
Stephenson [21]	2010	USA	1999–2007	Retrospective	Yes	Yes	No	No	Blind	3
Font [22]	2011	Spain	1991–2007	Retrospective	Yes	NA	No	No	NA	3
Kauffman [23]	2011	USA	2006–2008	Prospective	Yes	Yes	No	No	NA	4
Park(a) [24]	2011	Korea	1999–2009	Retrospective	Yes	NA	Yes	Yes	NA	5
Park(b) [25]	2011	Korea	1991–2008	Retrospective	Yes	Yes	No	No	NA	3
Gondo [26]	2012	Japan	2000–2009	Retrospective	Yes	Yes	No	Yes	NA	4
Otto [27]	2012	Germany	1989–2008	Retrospective	No	NA	Yes	Yes	NA	4
Afonso [28]	2013	Portugal	1996–2005	Retrospective	Yes	NA	Yes	No	NA	4
Eisenberg [29]	2013	USA	1980–2008	Retrospective	Yes	NA	Yes	No	Blind	5
Lotan [30]	2013	USA	2007–2012	Prospective	No	NA	No	No	NA	3

LVI: lymphovascular invasion, NA: not available.

### Study Characteristics

The main features of included studies are listed in Tables 1 and 2. The 21 studies had originated from the United States (9), Europe (5), Asia (6), and multiple countries (1). Two studies were based on a prospective cohort design. Four of these studies included <100 patients, and 11 studies had enrolled >200 patients. The median follow-up durations ranged from 18 months to 10.5 years, while three studies did not provide clear follow-up duration. All of the studies were published between 2007 and 2013. Other characteristics such as tumor characteristics and pathologic results are summarized in Table S1. Of the 12,527 patients included in the meta-alaysis, LVI was detected in 34.6% in radical cystectomy specimens. There were higher frequencies of LVI with higher pathological T stages and tumor grades, as well as lymph node metastasis (Table S2). Of the 35 survival analyses, 33 (94.3%) directly reported HRs or p-values with event number for multivariate analysis. In studies using multivariate analysis, the most common cofactors used to assess the risk of mortality was pathologic T stage (Table S3).

**Table 2:** Patient characteristics of the eligible studies.

Study	No. of patients	Median age, range (yr)	Gender (m/f)	Neoadjuvant chemotherapy	Adjuvant chemotherapy	Median FU, range (mon)
Turkolmez [10]	154	Primary MIBC: 59.8 (mean), NA Secondary MIBC: 60.3 (mean), NA	134/20	NA	NA	Primary MIBC: 77.8 (mean), NA Secondary MIBC: 90.3 (mean), NA
Canter [11]	356	65.5 (mean), NA	285/71	NA	NA	46.4 (mean), NA
Matsumoto [12]	92	63, 40–81	75/17	0	17	25.3, 1.1–196.1
Fairey [13]	468	66 (mean), NA	367/101	0	82	NA, NA
Streeper [14]	126	LVI -: 64.0 (mean), 44–87	101/25	16	41	LVI -: 1.66 yr, 0.25–10.16 yr
		LVI+: 64.8 (mean), 35–85				LVI+: 1.79 yr, 0.04–10.57 yr
Hugen [15]	260	No recurrence: 64.8 (mean), NA	193/67	NA	NA	NA, NA
		Recurrence: 68.4 (mean), NA				
Kim [16]	406	60.8, 27–79	360/46	0	0	66.3, 3–232
Ku [17]	155	60.2, 32–84	128/27	0	0	34.3, 1.0–162.4
Manoharan [18]	357	NA, NA	285/72	0	NA	NA, NA
Palmieri [19]	265	69, 46–93	218/47	0	0	108, 1–216
Shariat [20]	4257	67, NA	3373/864	0	954	43, 0.1–324.0
Stephenson [21]	134	68, 59–75	102/32	0	90	23, 10–36 (IQR)
Font [22]	57	64, 41–80	54/3	57	NA (RT: 5)	45, 13–190
Kauffman [23]	85	73.5, 41.4–93.8	67/18	17	10*	18 (mean), NA
Park(a) [24]	155	67.8, 38–80	127/28	0	0	36.6 (mean), 12–141
Park(b) [25]	450	pN-: 63, 38–85	408/42	0	86	26.8, 2–204
		pN+: 63, 37–80				
Gondo [26]	194	70, 38–85	162/32	0	48	26.8, 3.1–131.8
Otto [27]	2483	66.4, 60.1–72.5 (IQR)	1976/507	0	245	42, 21–79 (IQR)
Afonso [28]	81	71, 41–83	66/15	0	0	24, 1–132
Eisenberg [29]	1776	68, 62–75 (IQR)	1464/312	0	131 (RT: 17)	10.5 yr, 7.3–15.3 yr (IQR)
Lotan [30]	216	70, 62–76	171/15	48	29	20, 10–37 (IQR)

*chemotherapy or radiation therapy.

FU: follow-up, MIBC: muscle-invasive bladder cancer, NA: not available, LVI: lymphovascular invasion, IQR: interquartile range, RT: radiation therapy.

### Meta-analysis

**Figure 2:**
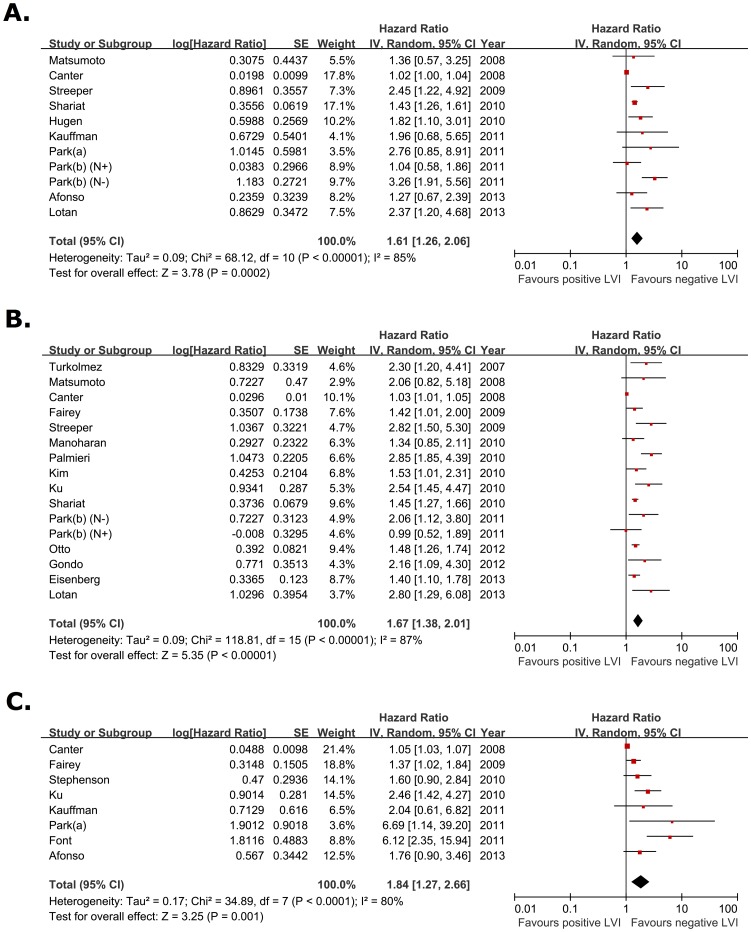
Forest plots of prognosis of lymphovascular invasion. The horizontal lines correspond to the study-specific hazard ration and 95% confidence interval, respectively. The area of the squares reflects the study-specific weight. The diamond represents the pooled results of hazard ratio and 95% confidence interval. (A) Recurrence-free survival. (B) Cancer-specific survival. (C) Overall survival.

According to a priori assumptions about the likelihood for heterogeneity between primary studies, the pooled HR estimate of the each study was calculated by the random effect model. Figure 2 demonstrates a forest plot of the individual HRs and results from the meta-analysis. When 10 eligible studies (11 dataset) were pooled into the meta-analysis for recurrence-free survival (RFS), we found that LVI was significantly associated with worse RFS (pooled HR, 1.61; 95% CI, 1.26–2.06; Z = 3.78). Cochrane Q test (Chi^2^ = 68.12; p<0.000001) and test of inconsistency (I^2^ = 85%) could not exclude a significant heterogeneity (Fig. 2a). The meta-analysis was performed on 15 studies (16 dataset) assessing the association of LVI and cancer-specific survival (CSS). The pooled HR was 1.67 (95% CI, 1.38–2.01; Z = 5.35) despite the heterogeneity among studies (p<0.00001 for heterogeneity test; I^2^ = 87%) (Fig. 2b). Eight studies provided data on overall survival (OS), and meta-analysis of OS suggested that LVI correlated with poor OS (pooled HR, 1.84; 95% CI, 1.27–2.66; Z = 3.25) with a large heterogeneity in the data (p<0.00001 for heterogeneity test; I^2^ = 80%) (Fig. 2c).

### Subgroup Analysis

Considering the substantial heterogeneity among the studies, we performed subgroup analyses to investigate if there were differences in results by publication year, the country of origin in which the study was conducted, number of patients, pathologic N stage, median follow-up, HR estimation, analysis results, and methodological quality scales (Table S4 to S6). The pooled HR for almost all subgroup analyses again supported LVI as a prognostic marker. However, the association between LVI and worse RFS did not remain statistically significant in studies of Asian subjects (pooled HR, 1.85; 95% CI, 0.98–3.51; Z = 1.89; p = 0.03 for heterogeneity test; I^2^ = 67). Notably, many of these subgroup analyses revealed heterogeneities of data.

### Sensitivity Analysis

We performed one-way sensitivity analyses by stepwise excluding a single study and calculating again the pooled HR for remaining studies. The pooled HRs and 95% CIs were not significantly altered when any one of the 21 studies was omitted, which indicated that no single study had a significant impact on the combined risk estimates and confirmed the robustness of the result of this meta-analysis. Omitting a certain study did not reduce inter-study heterogeneity significantly in the sensitivity analysis.

### Publication Bias

**Figure 3:**
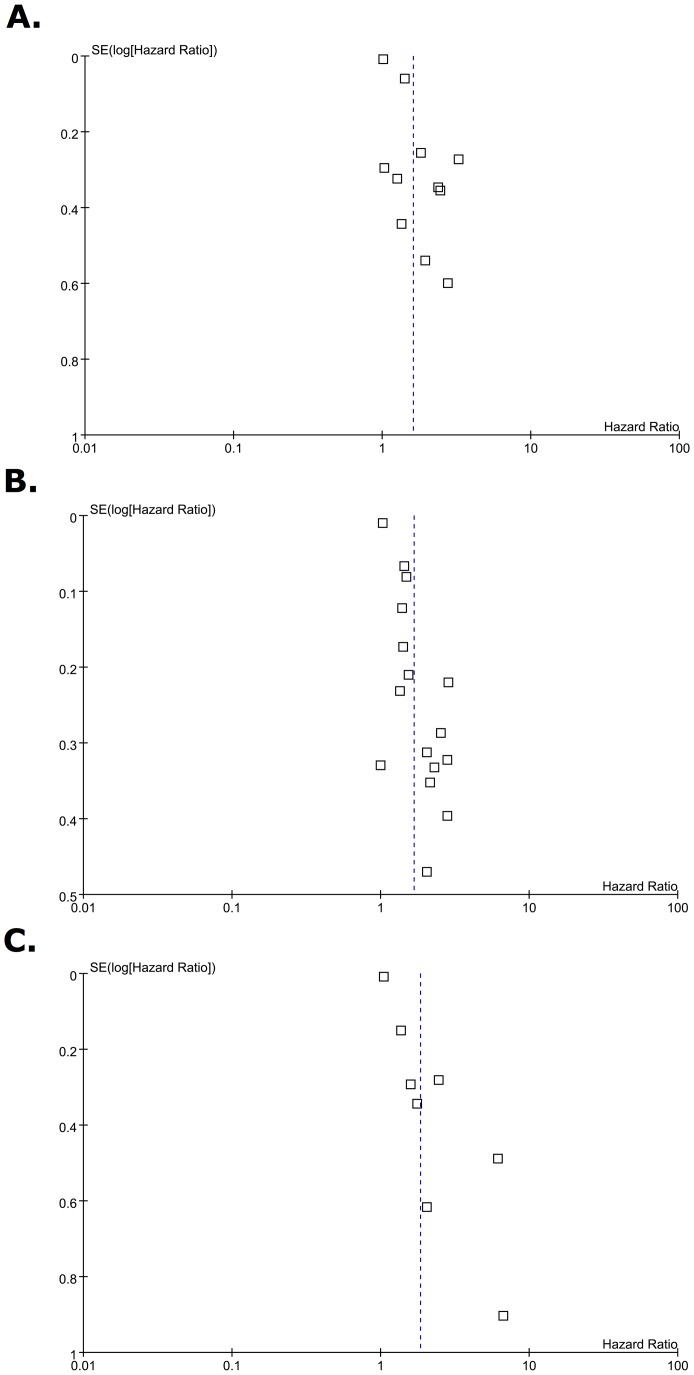
Begg’s Funnel plots for publication bias test. Each point represents a separate study for the indicated association. Vertical line represents the mean effects size. (A) Recurrence-free survival. (B) Cancer-specific survival. (C) Overall survival.

Begg’s funnel plot was used to examine publication bias (Fig. 3). No significant publication bias was found in the meta-analysis of survival outcome except for the association between LVI and OS. The funnel plot for OS demonstrated a certain degree of asymmetry, which suggested a slight publication bias. Begg’s test indicated no publication bias among these studies regarding HR of OS, CSS and OS with p values of 0.103, 0.6915 and 0.1021, respectively, but Egger’s test demonstrated a publication bias (all P<0.05). These results indicated a possibility that publication bias may have played a role in the observed effect.

## Discussion

Despite remarkable advances in treatment, the prognosis of bladder cancer remains unsatisafactory at the present time. Identification of the risk of disease recurrence and mortality in bladder cancer is critical to guide surveillance and select adjuvant therapies. Many studies have investigated potential prognostic factors for patients with bladder cancer, in order to guide therapeutic approaches and improve survival outcomes. LVI has been found in association with lymph node invasion, distant metastasis, and poor prognosis in patients with other sold tumors [31], [32]. Numerous studies have been performed to assess the prognostic value of LVI, but the results are still controversial and ambiguous in the management of bladder cancer.

To our knowledge, this meta-analysis is the first study to systemically assess the association between LVI and prognosis of bladder cancer. This study aggregated the outcomes of 12,527 bladder cancer patients who underwent radical cystectomy, as they were reported in 21 individual studies. We found that LVI was present in 34.6% of patients treated with radical cystectomy for bladder cancer. The significant associations were found between LVI and pathological parameters such as pathologic T stage, tumor grade, and pathologic N stage. Pooled analysis of the included studies found a significant correlation between LVI and poor survival outcome, suggesting that LVI is a significant predictor for poor survival in these patients. Sensitivity analysis demonstrated that omission of any single study did not have a significant impact on the combined risk estimates, further confirming the prognostic value of LVI in bladder cancer. Although subgroup analyses also demonstrated similar results, LVI was not significantly correlated with poor RFS for patients living in Asian countries. The characteristics of bladder cancer in different regions might differ because of diverse environmental and genetic factors, As such, the prognostic value of LVI in bladder cancer might differ across study locations. More studies with larger sample sizes in Asian countries are thus needed to further elucidate the prognostic value of LVI.

In addition to lymphatic metastasis, LVI is most likely associated with hematogenous tumor dissemination. Infiltration of the vascular and/or lymphatic structures by tumor cells is an important step in tumor dissemination [33]–[37]. Malignant cells invade the lymphovascular space, proliferate, and then permeate the local lymphatics or spread more widely [38]. This association is not limited to bladder cancer, and has also been shown in other cancers [39]–[41]. In addition, LVI is an important prognostic factor in various malignancies such as liver, testis, and penile cancer. In other malignancies [42], [43], LVI has been added the TNM staging system, allowing for improved cancer staging and treatment decision-making. Despite the increasing numbers of published studies that have added to the general knowledge about the prognostic role of LVI in bladder cancer, LVI is not a part of the TNM staging system or treatment guidelines for bladder cancer. Upstaging tumors on the basis of LVI might improve the accuracy of prognosis in bladder cancer, and therefore is a worthy consideration.

Several limitations of this meta-analysis should be acknowledged. One weakness of our study was publication bias, which could be seen from the publication bias evaluation of OS outcomes; the reported HR might be an overestimation of the true effect size. Because studies with negative results are less likely to be published than those representing positive data, and even if these results are published, they are more frequently reported in a brief way and not easily available for analysis, meta-analyses of selective reports may often introduce bias. It should be also noted that we could not exclude the bias associated with reviewing articles written in only the English language. Studies with positive results are more frequently published in English language, while studies with negative results tend to be published in the native language of respective authors [44].

The second limitation was heterogeneity. In sensitivity analysis, omission of any individual study did not reduce the heterogeneity. Our meta-analysis relied on publication but not on individual patient data. Studies have differed in baseline characteristics of patients. Though the random effect model takes heterogeneity into account and was used to analyze the studies with heterogeneities, the conclusion drawn in this meta-analysis should be approached with caution.

Moreover, we admit that meta-analysis of prognostic literature is associated with a number of inherent limitations. One of these key limitations is the general prevalence of retrospective study design in this setting. Only two studies included in the current meta-analysis specified a prospective design, with the remaining studies providing a lower level of evidence. There is a clear need for the initiation of a prospective multicenter trial to provide more definite answers.

In addition to these study limitation, significant differences in the assessment of prognostic factors have been observed among pathologists [45]. Because retraction artifacts in the surrounding stromal tissue can mimic vascular invasion, experts have recommended reporting LVI only in unequivocal cases, using immunohistochemistry if necessary [46]. However, the use of immunohistochemical staining to identify lymphatic vessels remains controversial and is not practical for everyday clinical use [47], [48]. It is of utmost importance that strict morphological criteria are established to standardize and render the diagnosis of LVI reproducible, allowing its recommendation in daily clinical settings [33]. In most studies on bladder cancer outcomes, vascular invasion and lymphatic invasion were combined as LVI. One of the reasons for this is that an unequivocal distinction between vascular invasion and lymphatic invasion is often difficult to make without the use of special stains, and that the clinical value of distinguishing vascular invasion from lymphatic invasion to predict bladder cancer outcomes has not been fully investigated. The development of novel markers and further studies are required to examine the significance of the distinction between blood and lymphatic vessels [26].

## Conclusions

This meta-analysis indicates that LVI is significantly associated with poorer outcomes in patients with bladder cancer who underwent radical cystectomy. LVI in radical cystectomy specimens not only predicts prognosis, but may also be useful in identifying a subgroup of patients who could benefit from adjuvant therapy. Strict criteria to unify the reproducibility of diagnosis as well as adequately designed prospective studies are required to provide a precise prognostic significance of LVI in bladder cancer.

## Supporting Information

Table S1 Tumor characteristics of the eligible studies.
(DOC)

Table S2 Lymphovascular invasion according to pathological features.
(DOC)

Table S3 Estimation of hazard ratio.
(DOC)

Table S4 Subgroup analysis for recurrence-free survival.
(DOC)

Table S5 Subgroup analysis for cancer-specific survival.
(DOC)

Table S6 Subgroup analysis for overall survival.
(DOC)

Checklist S1 PRISMA Checklist, page one.
(TIF)

Checklist S2 PRISMA Checklist, page two.
(TIF)
