# Supplementary material for: Prognostic Significance of Lymphovascular Invasion in Radical Cystectomy on Patients with Bladder Cancer: A Systematic Review and Meta-Analysis
Source: PLoS One. 2014 Feb 21;9(2):e89259. doi: 10.1371/journal.pone.0089259 (PMC3931717; doi:10.1371/journal.pone.0089259)
Supplement: Table S1 — Tumor characteristics of the eligible studies. (DOC) [file pone.0089259.s001.doc]

Table S1. Tumor characteristics of the eligible studies

| Study | Non-urothelial carcinoma | Pathologic T stage (T0/Tis/Ta/T1/T2/T3/T4) | Tumor grade (G0/G1/G2/G3) | Tumor grade (G0/LG/HG) | Concomitant CIS | Variant form | Positive surgical margin | Pathologic N stage (N0/N+) | Median no. of LNs removed |
| --- | --- | --- | --- | --- | --- | --- | --- | --- | --- |
| Turkolmez [10] | 0 | 0/0/0/0/86/41/27 | NA/NA/NA/NA | NA/NA/NA | NA | NA | NA | 134/20 | NA |
| Canter [11] | 0 | 23/53/4/29/85/112/50 | 23/NA/NA/NA | 23/NA/NA | NA | NA | NA | 257/99 | NA |
| Matsumoto [12] | 0 | 0/24 (TisTaT1)/68 (≥T2) | 0/46 (G1G2)/46 | 0/NA/NA | 15 | NA | NA | 72/20 | NA |
| Fairey [13] | 37 | 203 (≤T2)/238 (≥T3) | NA/NA/NA/NA | NA/NA/NA | 143 | NA | 55 | 316/108^*^ | NA |
| Streeper [14] | 0 | 11/0/0/9/13/33/60 | 11/NA/NA/NA | 11/NA/NA | 16 | NA | 18 | 126/0 | NA |
| Hugen [15] | NA | 169 (≤T2)/91 (≥T3) | NA/NA/NA/NA | NA/NA/NA | 166 | NA | 30 | 260/0 | 11 |
| Kim [16] | 0 | 0/0/137 (TaT1)/136/104/29 | 0/NA/NA/NA | 0/87/319 | 87 | 39 | 16 | 357/49 | NA |
| Ku [17] | 0 | 10/6/10/27/39/51/12 | 10/NA/NA/NA | 10/NA/NA | NA | NA | NA | NA/NA | NA |
| Manoharan [18] | 0 | 140 (≤T1)/84/98/35 | 0/49/15/293 | 0/NA/NA | 136 | NA | NA | 284/73 | NA |
| Palmieri [19] | 0 | 0/85 (TisT1)/55/82/43 | 0/15/35/215 | 0/NA/NA | NA | NA | NA | 204/61 | NA |
| Shariat [20] | 0 | 228/438/129/566/1012/1322/550 | 228/78/1761/2167 | 228/NA/NA | 2087 | NA | 266 | 3122/1071 | 18 |
| Stephenson [21] | 0 | NA/NA/NA/NA/NA/NA/NA | NA/NA/NA/NA | NA/NA/NA | NA | NA | 23 | 0/134 | 14 |
| Font [22] | 11 | 15/13 (TisT1)/11/5(≥T3) | 15/NA/NA/NA | 15/NA/NA | NA | NA | 5 | 48/9 | NA |
| Kauffman [23] | 0 | 12/11/7/12/12/26/6 | 12/NA/NA/NA | 12/NA/NA | 40 | 12 | 5 | 72/13 | 17 |
| Park(a) [24] | NA | 116 (≤T2)/39 (≥T3) | 0/2/65/88 | 0/NA/NA | 41 | NA | 2 | 155/0 | 14.9 (mean) |
| Park(b) [25] | 0 | 0/108 (TisTaT1)/146/143/53 | 0/6/59/385 | 0/NA/NA | NA | NA | NA | 321/129 | N0: 17, N+: 19 |
| Gondo [26] | 47 | 62 (≤T1)/46/58/28 | 0/21 (G1G2)/173 | 0/NA/NA | NA | NA | 20 | 173/21 | NA |
| Otto [27] | 0 | 708 (≤T1)/245/841/265 | 0/829 (G1G2)/1654 | 0/NA/NA | 765 | NA | NA | 1843/640 | 14 |
| Afonso [28] | 0 | 0/4/16(TaT1)/61 (≥T2) | 0/0/25/56 | 0/NA/NA | NA | 0 | NA | NA/NA | NA |
| Eisenberg [29] | 0 | 924 (≤T1)/307/474/71 | NA/NA/NA/NA | NA/NA/NA | NA | 128 | 25 | 1530/246 | NA |
| Lotan [30] | 0 | 8/0/0/78/43/59/28 | 8/NA/NA/NA | 8/2/206 | 96 | NA | 16 | 161/55 | 23 |

^*^not available (n = 44)

LG: low grade, HG: high grade, CIS: carcinoma in situ, LN: lymph node, NA: not available.
