# Supplementary material for: Prognostic Significance of Lymphovascular Invasion in Radical Cystectomy on Patients with Bladder Cancer: A Systematic Review and Meta-Analysis
Source: PLoS One. 2014 Feb 21;9(2):e89259. doi: 10.1371/journal.pone.0089259 (PMC3931717; doi:10.1371/journal.pone.0089259)
Supplement: Table S2 — Lymphovascular invasion according to pathological features. (DOC) [file pone.0089259.s002.doc]

Table S2. Lymphovascular invasion according to pathological features

| Study | No. (%) of positive LVI | T stage | | | | Tumor grade | | | | | Pathologic N stage | |
| --- | --- | --- | --- | --- | --- | --- | --- | --- | --- | --- | --- | --- |
|  |  | ≤T1 | T2 | T3 | T4 | G1 | G2 | G3 | LG | HG | pN0 | pN+ |
| Turkolmez [10] | NA | NA | NA | NA | NA | NA | NA | NA | NA | NA | NA | NA |
| Canter [11] | 114/356 (32.0) | 9/109 | 21/85 | 57/112 | 27/50 | NA | NA | NA | NA | NA | NA | NA |
| Matsumoto [12] | 45/92 (48.9) | NA | NA | NA | NA | NA | NA | NA | NA | NA | NA | NA |
| Fairey [13] | 150/468 (32.1) | NA | NA | NA | NA | NA | NA | NA | NA | NA | NA | NA |
| Streeper [14] | 94/126 (74.6) | 10/20 | 9/13 | 24/33 | 51/60 | NA | NA | NA | NA | NA | 94/126 | 0/0 |
| Hugen [15] | 67/260 (25.8) | NA | NA | NA | NA | NA | NA | NA | NA | NA | 67/260 | 0/0 |
| Kim [16] | 343/406 (84.5) | NA | NA | NA | NA | NA | NA | NA | NA | NA | NA | NA |
| Ku [17] | 103/155 (66.5) | NA | NA | NA | NA | NA | NA | NA | NA | NA | NA | NA |
| Manoharan [18] | 105/357 (29.4) | 6/140 | 34/84 | 48/98 | 17/35 | 0/49 | 3/15 | 102/293 | NA | NA | 61/284 | 44/73 |
| Palmieri [19] | 77/265 (29.1) | 5/85 | 15/55 | 30/82 | 27/43 | 1/15 | 8/35 | 68/215 | NA | NA | 39/204 | 38/61 |
| Shariat [20] | 1407/4257 (33.1) | 62/1361 | 317/1012 | 691/1322 | 335/550 | 4/78 | 673/1761 | 727/2167 | NA | NA | 702/3122 | 693/1071 |
| Stephenson [21] | 87/134 (64.9) | NA | NA | NA | NA | NA | NA | NA | NA | NA | 0/0 | 87/134 |
| Font [22] | 9/57 (15.8) | NA | NA | NA | NA | NA | NA | NA | NA | NA | NA | NA |
| Kauffman [23] | 14/85 (16.5) | NA | NA | NA | NA | NA | NA | NA | NA | NA | NA | NA |
| Park(a) [24] | 57/155 (36.8) | NA | NA | NA | NA | NA | NA | NA | NA | NA | 57/155 | 0/0 |
| Park(b) [25] | 188/450 (41.8) | NA | NA | NA | NA | NA | NA | NA | NA | NA | 93/321 | 95/129 |
| Gondo [26] | 99/194 (51.0) | NA | NA | NA | NA | NA | NA | NA | NA | NA | NA | NA |
| Otto [27] | 876/2483 (35.3) | NA | NA | NA | NA | NA | NA | NA | NA | NA | NA | NA |
| Afonso [28] | 37/81 (45.7) | NA | NA | NA | NA | 0/0 | NA | NA | NA | NA | NA | NA |
| Eisenberg [29] | 339/1776 (19.1) | NA | NA | NA | NA | NA | NA | NA | NA | NA | NA | NA |
| Lotan [30] | 73/216 (33.8) | NA | NA | NA | NA | NA | NA | NA | NA | NA | NA | NA |
| Total | 4284/12373 (34.6) | 92/1715 | 396/1249 | 850/1647 | 457/738 | 5/142 | 684/1811 | 897/2675 | NA | NA | 1113/4472 | 957/1468 |

LVI: lymphovascular invasion, LG: low grade, HG: high grade, NA: not available.
