# Supplementary material for: Prognostic Significance of Lymphovascular Invasion in Radical Cystectomy on Patients with Bladder Cancer: A Systematic Review and Meta-Analysis
Source: PLoS One. 2014 Feb 21;9(2):e89259. doi: 10.1371/journal.pone.0089259 (PMC3931717; doi:10.1371/journal.pone.0089259)
Supplement: Table S3 — Estimation of hazard ratio. (DOC) [file pone.0089259.s003.doc]

**Table S3**. Estimation of the hazard ratio

| Study | Survival analysis | HR estimation | Co-factors | Analysis results |
| --- | --- | --- | --- | --- |
| Turkolmez [10] | CSS | HR, 95% CI | Age, gender, pT stage, tumor grade, pN stage, metastasis during follow-up | Significant |
| Canter [11] | RFS | HR, 95% CI | Age, pT stage, pN stage | Not significant |
|  | CSS | HR, 95% CI | Age, pT stage, pN stage | Significant |
|  | OS | HR, 95% CI | Age, pT stage, pN stage | Significant |
| Matsumoto [12] | RFS | HR, 95% CI | pT stage, tumor grade, pN stage, uroplakin III expression | Not significant |
|  | CSS | HR, 95% CI | pT stage, tumor grade, pN stage, uroplakin III expression | Not significant |
| Fairey [13] | CSS | HR, 95% CI | Age, comorbidity, pT stage, pN stage, margin status, no. of LNs removed, adjuvant chemotherapy, surgeon procedure volume | Significant |
|  | OS | HR, 95% CI | Age, comorbidity, pT stage, pN stage, margin status, no. of LNs removed, adjuvant chemotherapy, surgeon procedure volume | Significant |
| Streeper [14] | RFS | P value, event no. (univariate) | - | Significant |
|  | CSS | HR, event no. | Clinical stage, chemotherapy | Significant |
| Hugen [15] | RFS | P value, event no. | pT stage, perineural invasion, margin status, no. of LNs removed | Significant |
| Kim [16] | CSS | HR, 95% CI | Age, hydronephrosis, hydronephrosis grade, pT stage, pN stage, margin status, no. of LNs removed | Significant |
| Ku [17] | CSS | HR, 95% CI | Age, gender, ASA score, no. of previous TUR, history of intravesical BCG instillation, clinical stage, type of procedure, perineural invasion | Significant |
|  | OS | HR, 95% CI | Age, gender, ASA score, no. of previous TUR, history of intravesical BCG instillation, clinical stage, type of procedure, perineural invasion | Significant |
| Manoharan [18] | CSS | HR, 95% CI | pT stage, tumor grade, pN stage | Not significant |
| Palmieri [19] | CSS | HR, 95% CI (univariate) | - | Significant |
| Shariat [20] | RFS | HR, 95% CI | pT stage, tumor grade, margin status, pN stage, no. of LNs removed, adjuvant chemotherapy | Significant |
|  | CSS | HR, 95% CI | pT stage, tumor grade, margin status, pN stage, no. of LNs removed, adjuvant chemotherapy | Significant |
| Stephenson [21] | OS | HR, 95% CI | Charlson comorbidity index, pT stage, margin status, aggregate LN metastasis diameter, LN density, extranodal extension | Not significant |
| Font [22] | OS | HR, 95% CI | Age, hydronphrosis, clinical stage, resection, pT stage, variant form, chemotherapy regimen, BRCA1 expression | Significant |
| Kauffman [23] | RFS | HR, 95% CI | pT stage, LN density | Not significant |
|  | OS | HR, 95% CI | pT stage, LN density | Not significant |
| Park(a) [24] | RFS | HR, 95% CI | Tumor grade (2004 WHO), perineural invasion, no. of LNs removed | Not significant |
|  | OS | HR, 95% CI | Tumor grade (2004 WHO), perineural invasion, no. of LNs removed | Significant |
| Park(b) pN- [25] | RFS | HR, 95% CI | Age, gender, pT stage, tumor grade, concomitant CIS, no. of LNs removed | Significant |
|  | CSS | HR, 95% CI | Age, gender, pT stage, tumor grade, concomitant CIS, no. of LNs removed | Significant |
| Park (b) pN+ [25] | RFS | HR, 95% CI | Age, gender, pT stage, tumor grade, concomitant CIS, no. of LNs removed, LN density, adjuvant chemotherapy | Not significant |
|  | CSS | HR, 95% CI | Age, gender, pT stage, tumor grade, concomitant CIS, no. of LNs removed, LN density, adjuvant chemotherapy | Not significant |
| Gondo [26] | CSS | HR, 95% CI | pT stage, margin status | Significant |
| Otto [27] | CSS | HR, 95% CI | Age, gender, pT stage, tumor grade, concomitant CIS, pN stage, no. of LNs removed, adjuvant chemotherapy, time period | Significant |
| Afonso [28] | RFS | HR, 95% CI | pT stage, tumor grade, loco-regional metastasis, embolic blood vessels invasion, RKIP expression | Not significant |
|  | OS | HR, 95% CI | pT stage, tumor grade, loco-regional metastasis, embolic blood vessels invasion, RKIP expression | Not significant |
| Eisenberg [29] | CSS | HR, 95% CI | Charlson index, ECOG status, hydronphrosis, current smoker, pT stage, multifocal invasive disease, adjuvant chemotherapy | Significant |
| Lotan [30] | RFS | HR, 95% CI | pT stage, margin status, pN stage, adjuvant chemotherapy, no. of altered biomarkers | Significant |
|  | CSS | HR, 95% CI | pT stage, margin status, pN stage, adjuvant chemotherapy, no. of altered biomarkers | Significant |

HR: hazard ratio, OS: overall survival, CI: confidence interval, CIS: carcinoma in situ, LN: lymph node, CSS: cancer-specific survival, RFS: recurrence-free survival, ASA: American Society of Anesthesiologists, TUR: transurethral resection, BCG: bacillus Calmette-Guerin, ECOG: Eastern Cooperative Oncology Group.
