# Supplementary material for: Prognostic Significance of Lymphovascular Invasion in Radical Cystectomy on Patients with Bladder Cancer: A Systematic Review and Meta-Analysis
Source: PLoS One. 2014 Feb 21;9(2):e89259. doi: 10.1371/journal.pone.0089259 (PMC3931717; doi:10.1371/journal.pone.0089259)
Supplement: Table S4 — Subgroup analysis for recurrence-free survival. (DOC) [file pone.0089259.s004.doc]

**Table S4**. Subgroup analysis for recurrence-free survival

|  | No. of included articles | No. of cases | Pooled HR (95% CI) | Chi^2^ (p value) | I^2^ |
| --- | --- | --- | --- | --- | --- |
| Publication year |  |  |  |  |  |
| 2007-2010 | 5 | 5091 | 1.41 (1.06-1.87) | 39.95 (<0.00001) | 90% |
| 2011-2013 | 5 (6 dataset) | 987 | 1.89 (1.24-2.89) | 10.46 (0.06) | 52% |
| Region |  |  |  |  |  |
| Asia | 3 (4 dataset) | 697 | 1.85 (0.98-3.51) | 9.05 (0.03) | 67% |
| Others | 7 | 5381 | 1.50 (1.15-1.95) | 47.13 (<0.00001) | 87% |
| No. of patients |  |  |  |  |  |
| <200 | 6 | 668 | 1.51 (1.11-2.06) | 5.04 (0.41) | 1% |
| ≥200 | 5 | 5410 | 1.64 (1.19-2.25) | 57.33 (<0.00001) | 93% |
| Pathologic N stage |  |  |  |  |  |
| pN- | 6 | 4241 | 1.94 (1.55-2.44) | 6.98 (0.22) | 28% |
| pN+ | 1 | 129 | 1.04 (0.58-1.86) | Not applicable | Not applicable |
| Median follow-up |  |  |  |  |  |
| <60 months | 9 (10 dataset) | 5818 | 1.58 (1.22-2.05) | 63.26 (<0.00001) | 86% |
| ≥60 months | 0 | 0 | Not applicable | Not applicable | Not applicable |
| HR estimation |  |  |  |  |  |
| Univariate | 1 | 126 | 2.45 (1.22-4.92) | Not applicable | Not applicable |
| Multivariate | 9 (10 dataset) | 5952 | 1.55 (1.21-1.99) | 62.23 (<0.00001) | 86% |
| Analysis results |  |  |  |  |  |
| Not significant | 5 | 898 | 1.03 (0.96-1.11) | 5.09 (0.4) | 2% |
| Significant | 5 | 5180 | 2.05 (1.42-2.96) | 12.81 (0.01) | 69% |
| Quality scale |  |  |  |  |  |
| <4 | 5 (6 dataset) | 1408 | 1.76 (1.10-2.91) | 35.18 (<0.00001) | 86% |
| ≥4 | 5 | 4670 | 1.43 (1.28-1.61) | 1.7 (0.79) | 0% |

HR: hazard ratio, CI: confidence interval, LVI: lymphovascular invasion, ELCWP: European Lung Cancer Working Party.
