# Supplementary material for: Prognostic Significance of Lymphovascular Invasion in Radical Cystectomy on Patients with Bladder Cancer: A Systematic Review and Meta-Analysis
Source: PLoS One. 2014 Feb 21;9(2):e89259. doi: 10.1371/journal.pone.0089259 (PMC3931717; doi:10.1371/journal.pone.0089259)
Supplement: Table S5 — Subgroup analysis for cancer-specific survival. (DOC) [file pone.0089259.s005.doc]

**Table S5**. Subgroup analysis for cancer-specific survival

|  | No. of included articles | No. of cases | Pooled HR (95% CI) | Chi^2^ (p value) | I^2^ |
| --- | --- | --- | --- | --- | --- |
| Publication year |  |  |  |  |  |
| 2007-2010 | 10 | 6636 | 1.71 (1.33-2.18) | 80.74 (<0.00001) | 89% |
| 2011-2013 | 5 (6 dataset) | 5119 | 1.53 (1.29-1.82) | 6.52 (0.26) | 23% |
| Region |  |  |  |  |  |
| Asia | 5 (6 dataset) | 1297 | 1.77 (1.36-2.31) | 5.83 (0.32) | 14% |
| Others | 10 | 10458 | 1.61 (1.30-2.00) | 95.28 (<0.00001) | 91% |
| No. of patients |  |  |  |  |  |
| <200 | 6 | 850 | 2.06 (1.51-2.81) | 6.54 (0.26) | 24% |
| ≥200 | 10 | 10905 | 1.54 (1.25-1.88) | 88.15 (<0.00001) | 90% |
| Pathologic N stage |  |  |  |  |  |
| pN- | 6 | 4314 | 1.76 (1.51-2.05) | 3.09 (0.69) | 0% |
| pN+ | 1 | 129 | 0.99 (0.52-1.89) | Not applicable | Not applicable |
| Median follow-up |  |  |  |  |  |
| ≤60 months | 9 (10 dataset) | 8329 | 1.65 (1.30-2.10) | 80.01 (<0.00001) | 89% |
| >60 months | 4 | 2601 | 1.85 (1.30-2.64) | 9.03 (0.03) | 67% |
| HR estimation |  |  |  |  |  |
| Univariate | 1 | 265 | 2.85 (1.85-4.39) | Not applicable | Not applicable |
| Multivariate | 14 (15 dataset) | 11490 | 1.59 (1.33-1.91) | 98.42 (<0.00001) | 86% |
| Analysis results |  |  |  |  |  |
| Not significant | 3 | 578 | 1.31 (0.93-1.84) | 1.65 (0.44) | 0% |
| Significant | 13 | 11177 | 1.73 (1.41-2.13) | 115.67 (<0.00001) | 90% |
| Quality scale |  |  |  |  |  |
| <4 | 8 (9 dataset) | 2533 | 1.58 (1.20-2.08) | 34.91 (<0.0001) | 77% |
| ≥4 | 7 | 9222 | 1.68 (1.41-2.00) | 13.73 (0.03) | 56% |

HR: hazard ratio, CI: confidence interval, LVI: lymphovascular invasion, ELCWP: European Lung Cancer Working Party.
