# Supplementary material for: Prognostic Significance of Lymphovascular Invasion in Radical Cystectomy on Patients with Bladder Cancer: A Systematic Review and Meta-Analysis
Source: PLoS One. 2014 Feb 21;9(2):e89259. doi: 10.1371/journal.pone.0089259 (PMC3931717; doi:10.1371/journal.pone.0089259)
Supplement: Table S6 — Subgroup analysis for overall survival. (DOC) [file pone.0089259.s006.doc]

**Table S6**. Subgroup analysis for overall survival

|  | No. of included articles | No. of cases | Pooled HR (95% CI) | Chi^2^ (p value) | I^2^ |
| --- | --- | --- | --- | --- | --- |
| Publication year |  |  |  |  |  |
| 2007-2010 | 4 | 1113 | 1.43 (1.02-2.00) | 14.32 (0.003) | 79% |
| 2011-2013 | 4 | 378 | 3.06 (1.49-6.28) | 6.56 (0.14) | 46% |
| Region |  |  |  |  |  |
| Asia | 2 | 310 | 2.81 (1.45-5.47) | 1.12 (0.29) | 11% |
| Others | 6 | 1181 | 1.60 (1.12-2.29) | 21.56 (0.0006) | 77% |
| No. of patients |  |  |  |  |  |
| <200 | 6 | 667 | 2.40 (1.60-3.60) | 7.64 (0.18) | 35% |
| ≥200 | 2 | 824 | 1.15 (0.90-1.47) | 3.11 (0.08) | 68% |
| Pathologic N stage |  |  |  |  |  |
| pN- | 2 | 421 | 2.52 (0.70-9.05) | 2.32 (0.13) | 57% |
| pN+ | 1 | 134 | 1.60 (0.90-2.84) | Not applicable | Not applicable |
| Median follow-up |  |  |  |  |  |
| ≤60 months | 7 | 1023 | 2.11 (1.26-3.52) | 31.85 (<0.00001) | 81% |
| >60 months | 0 | 0 | Not applicable | Not applicable | Not applicable |
| HR estimation |  |  |  |  |  |
| Univariate | 0 | 0 | Not applicable | Not applicable | Not applicable |
| Multivariate | 8 | 1491 | 1.84 (1.27-2.66) | 34.89 (<0.00001) | 80% |
| Analysis results |  |  |  |  |  |
| Not significant | 3 | 300 | 1.71 (1.13-2.58) | 0.14 (0.93) | 0% |
| Significant | 5 | 1191 | 1.96 (1.20-3.19) | 29.48 (<0.00001) | 86% |
| Quality scale |  |  |  |  |  |
| <4 | 4 | 1015 | 1.55 (1.02-2.36) | 18.16 (0.0004) | 83% |
| ≥4 | 4 | 476 | 2.27 (1.53-3.35) | 2.09 (0.55) | 0% |

HR: hazard ratio, CI: confidence interval, LVI: lymphovascular invasion, ELCWP: European Lung Cancer Working Party.
