# Supplementary figures and images for: Prognostic Significance of Lymphovascular Invasion in Radical Cystectomy on Patients with Bladder Cancer: A Systematic Review and Meta-Analysis
Source: PLoS One. 2014 Feb 21;9(2):e89259. doi: 10.1371/journal.pone.0089259 (PMC3931717; doi:10.1371/journal.pone.0089259)

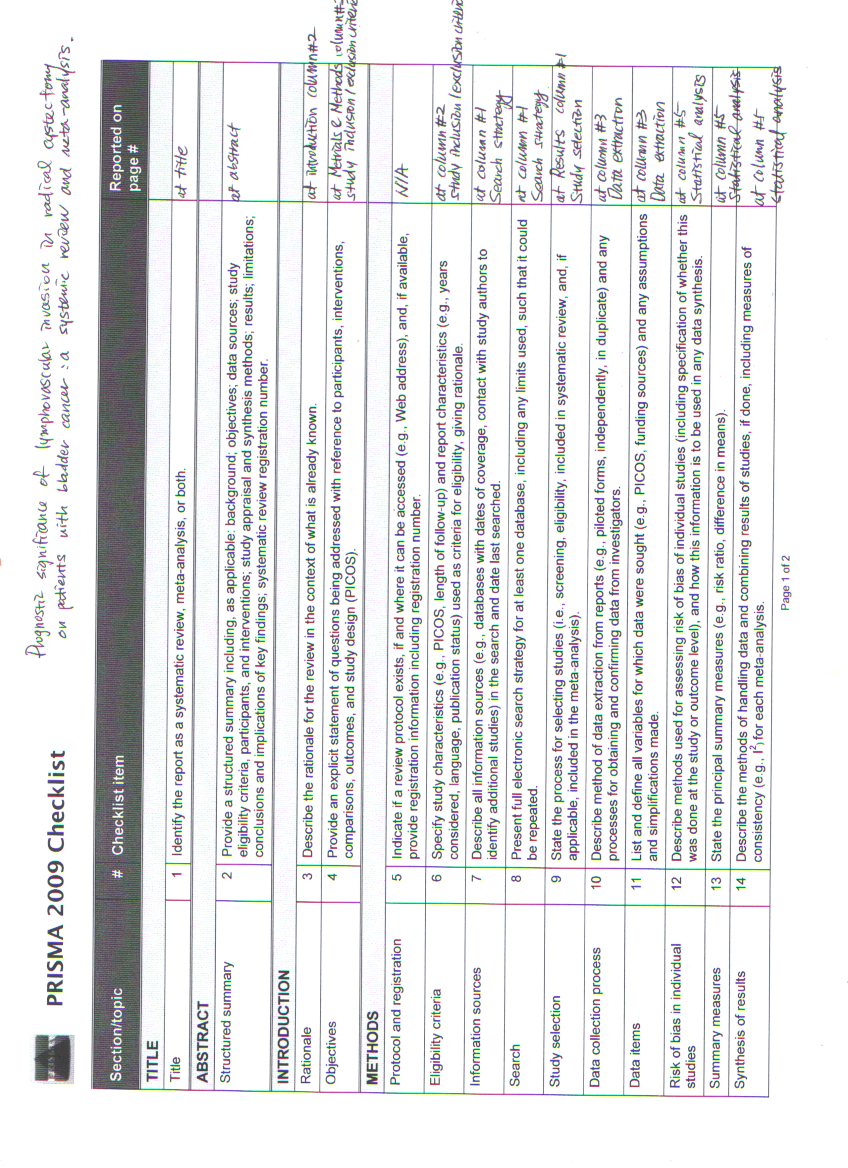

Supplement: Checklist S1 — PRISMA Checklist, page one. (TIF) [file pone.0089259.s007.tif]

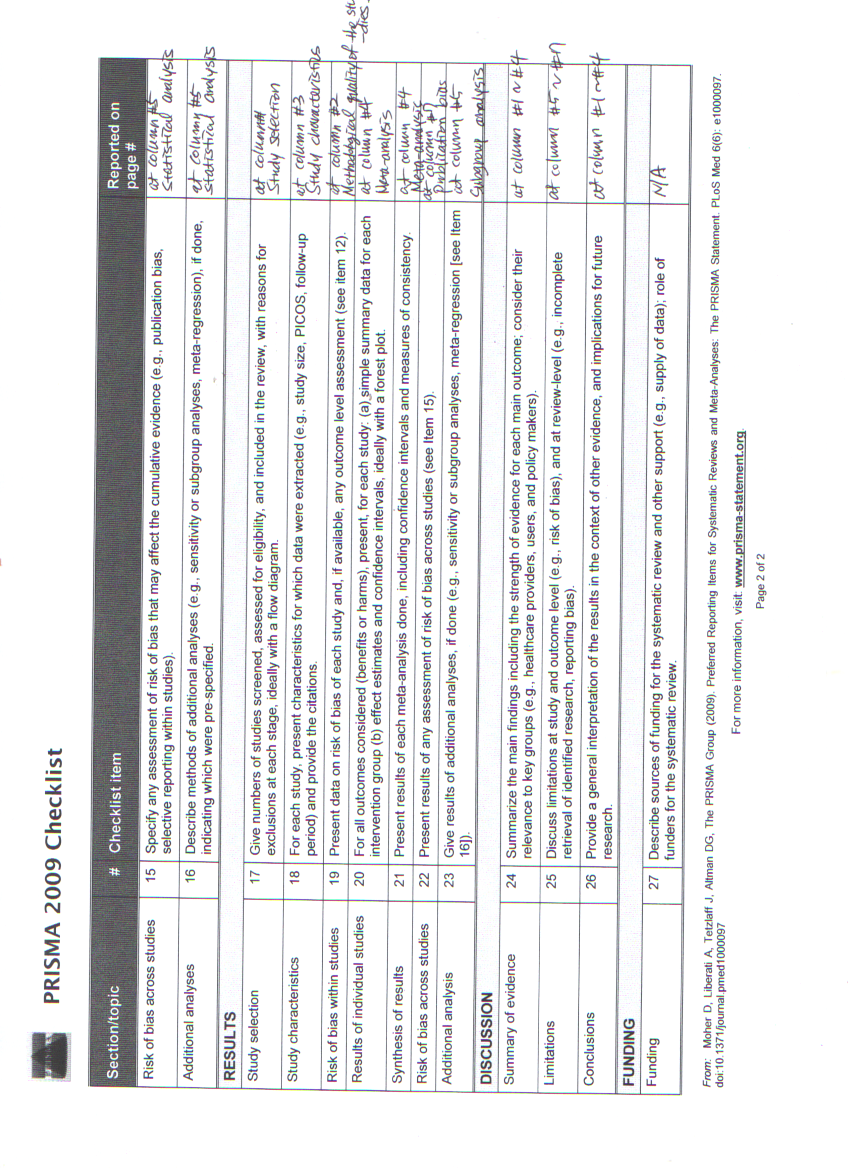

Supplement: Checklist S2 — PRISMA Checklist, page two. (TIF) [file pone.0089259.s008.tif]
